# Supplementary material for: Metabolomics Reveals the Impact of Overexpression of Cytosolic Fructose-1,6-Bisphosphatase on Photosynthesis and Growth in Nannochloropsis gaditana
Source: Int J Mol Sci. 2024 Jun 20;25(12):6800. doi: 10.3390/ijms25126800 (PMC11204352; doi:10.3390/ijms25126800)
Supplement: Supplementary file 1 [file ijms-25-06800-s001.zip › ijms-3021227-supplementary.pdf]

Supplementary Data

Table S1. Species information for cyFBPase from diverse photosynthetic eukaryotes

Species	Name
Land plants	*Arabidopsis thaliana*<br>*Nicotiana tabacum*
Chlorophyta	*Chlamydomonas reinhardtii*<br>*Nannochloropsis gaditana*
Bacillariophyta	*Fragilariopsis cylindrus*<br>*Fistulifera solaris*<br>*Phaeodactylum tricornutum*
Chrysophyta	*Aureococcus anophagefferens*<br>*Emiliania huxleyi*<br>*Pelagophyceae sp.*
Cyanobacteria	*Cyanobacterium sp.*
Euglenozoa	*Eutreptia viridis*<br>*Discoplastis spathirhyncha*<br>*Euglena gracilis*
Eumycophyta	*Saccharomyces cerevisiae*
Haptophyta	*Pavlovales sp.*<br>*Chrysochromulina tobinii*<br>*Diacronema lutheri*
Oomycota	*Globisporangium splendens*<br>*Lagenidium giganteum*<br>*Phytophthora sojae*<br>*Plasmopara halstedii*<br>*Pythium brassicae*<br>*Pythium insidiosum*<br>*Pythium oligandrum*
Pyrrophyta	*Symbiodinium sp.*

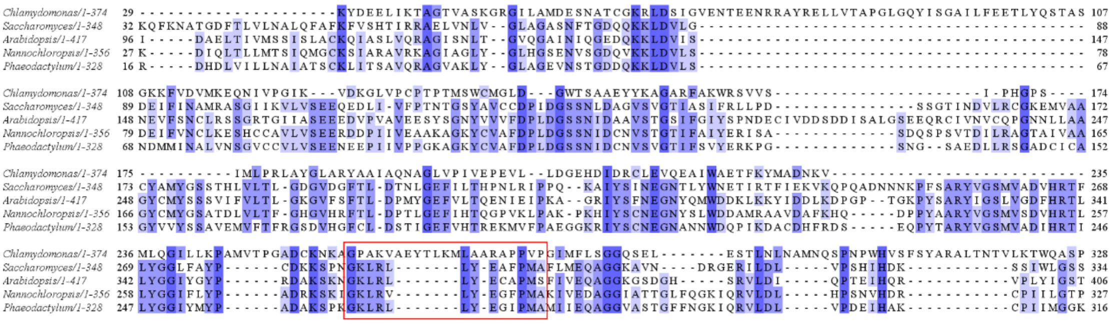

Figure S1. Multi-sequence alignment analysis. Red boxes are active areas. Analysis using InterPro (InterPro (ebi.ac.uk)).

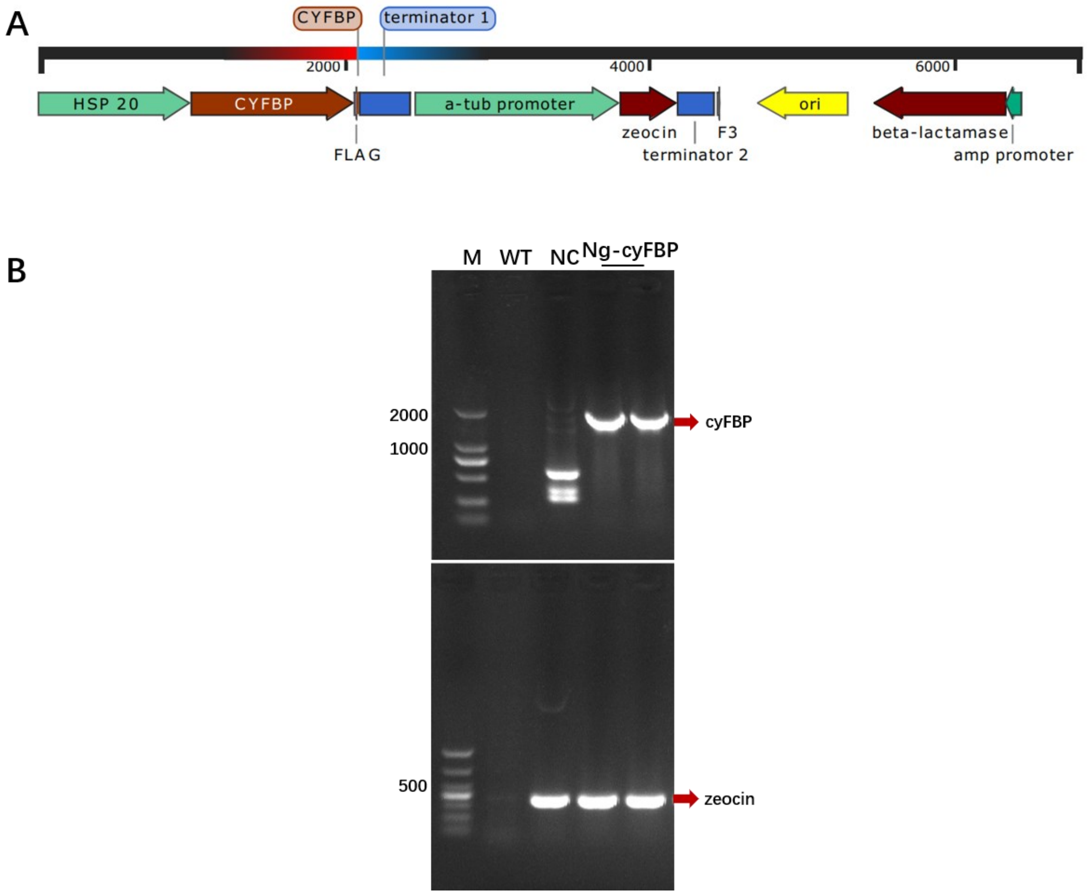

Figure S2. (A) overexpression vectors of Ng-cyFBP. (B) Electrophoretic verification results of the *cyFBP* and zeocin fragments derived from the transformed *N. gaditana* genome. NC: negative control (A vector lacking the target gene *cyFBPase*, containing only the zeocin resistance gene, to serve as a negative control)

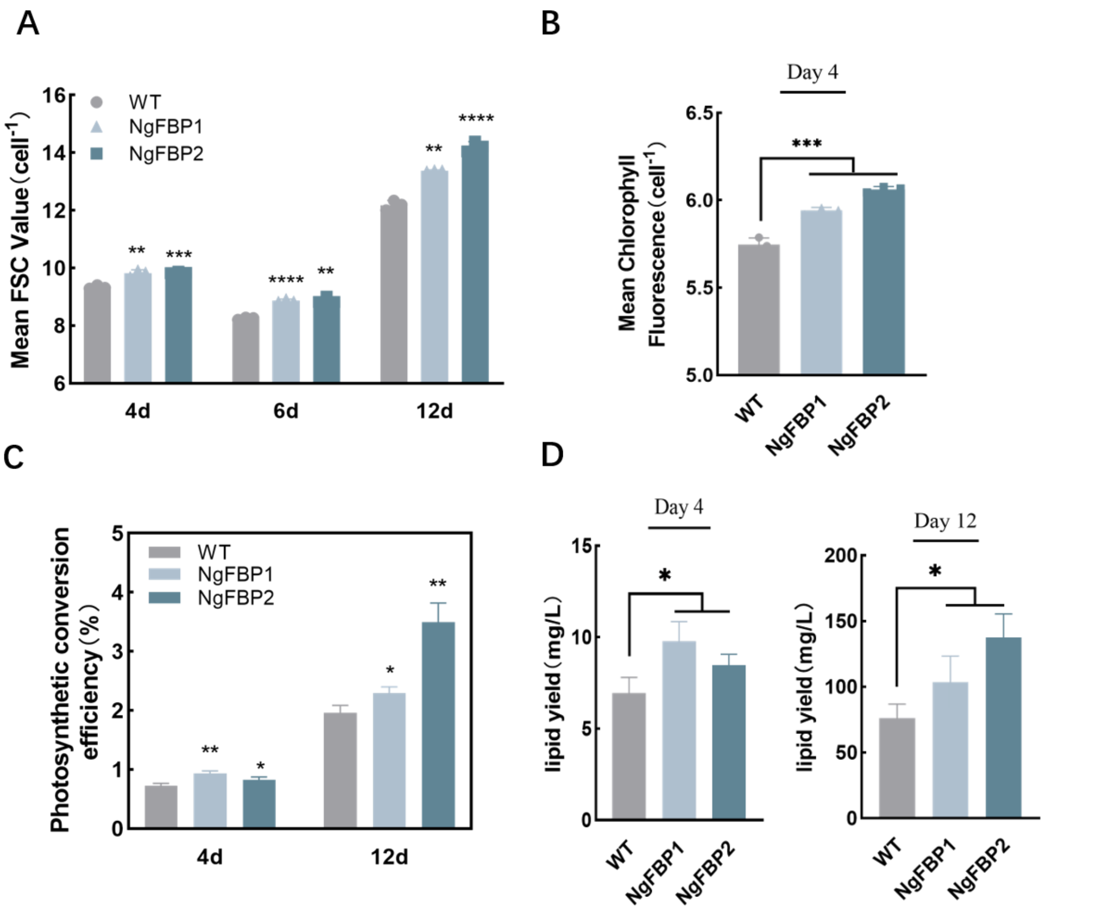

Figure S3. (A) FSC Value of the WT and NgFBP. (B) Chlorophyll fluorescence of WT and NgFBP. (C) Photosynthetic conversion efficiency of NgFBP. (D)Lipid yield on day 4 and day 12. ns indicates $p > 0.05$, * $p < 0.05$, ** $p < 0.01$, *** $p <0.001$, **** $p < 0.0001$. Error bars represent standard deviations calculated from three independent biological replicates. All data were analyzed through two-way ANOVA (GraphPad Prism).

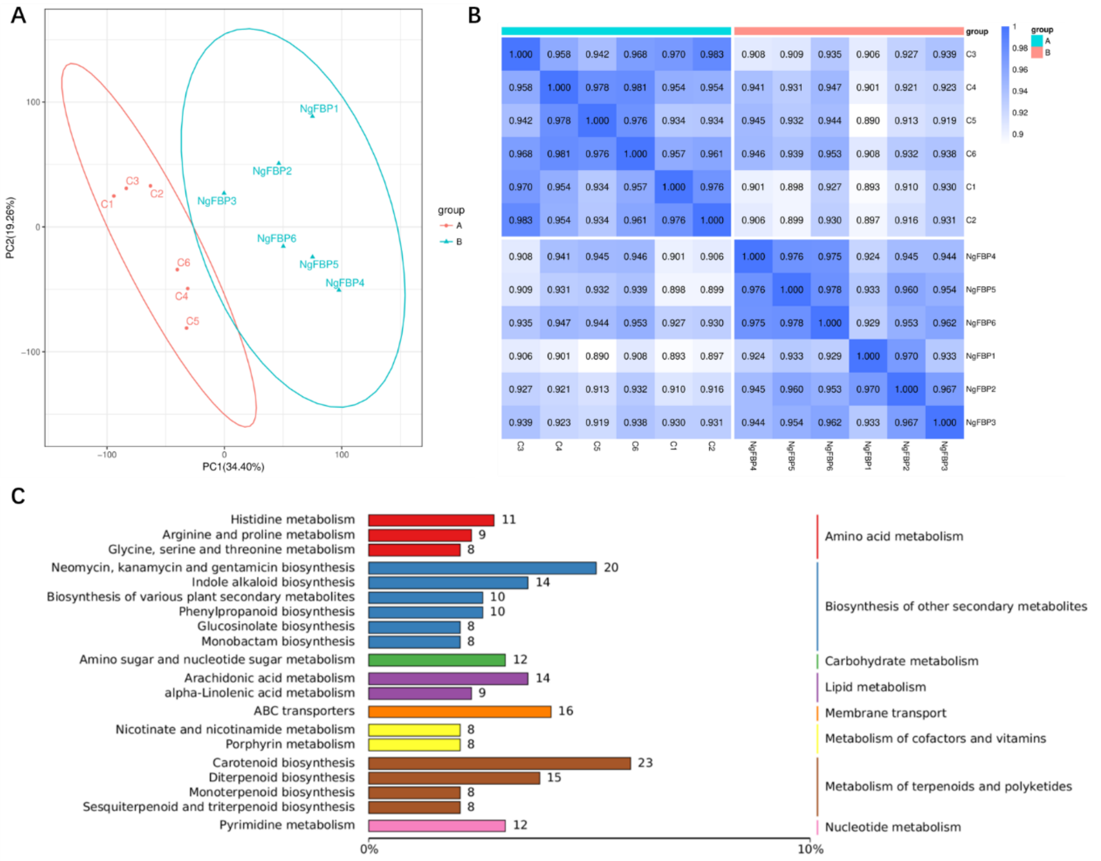

Figure S4. (A) Principal Component Analysis (PCA). (B) Correlation analysis of WT and NgFBP. (C) KEGG enrichment analysis of differentially metabolites in WT and NgFBP on day 4.
